# Organising health care services for people with an acquired brain injury: an overview of systematic reviews and randomised controlled trials

**DOI:** 10.1186/1472-6963-14-397

**Published:** 2014-09-17

**Authors:** Kate Laver, Natasha A Lannin, Peter Bragge, Peter Hunter, Anne E Holland, Emma Tavender, Denise O’Connor, Fary Khan, Robert Teasell, Russell Gruen

**Affiliations:** Occupational Therapy, La Trobe University, Melbourne, VIC Australia; Occupational Therapy, Alfred Health, Melbourne, VIC Australia; National Trauma Research Institute, Monash University and The Alfred Hospital, Kragujevac, VIC Australia; Rehabilitation, Aged and Community Care, Alfred Health, Melbourne, VIC Australia; Physiotherapy, La Trobe University, Melbourne, VIC Australia; Physiotherapy, Alfred Health, Melbourne, VIC Australia; Australian Satellite of the Cochrane Effective Practice and Organisation of Care (EPOC) Group’, Monash University, Melbourne, Australia; School of Public Health and Preventive Medicine, Monash University, Melbourne, Australia; Department of Rehabilitation Medicine, The University of Melbourne, Melbourne, Australia; Rehabilitation Medicine, Royal Melbourne Hospital, Melbourne, Australia; Physical Medicine and Rehabilitation, University of Western, Ontario, ON Canada

**Keywords:** Brain injuries, Craniocerebral trauma, Stroke, Systematic review, Rehabilitation, Case management, Delivery of health care, Quality assurance (Health Care)

## Abstract

**Background:**

Acquired brain injury (ABI) is the leading cause of disability worldwide yet there is little information regarding the most effective way to organise ABI health care services. The aim of this review was to identify the most up-to-date high quality evidence to answer specific questions regarding the organisation of health care services for people with an ABI.

**Methods:**

We conducted a systematic review of English papers using MEDLINE, EMBASE, PsycINFO, CINAHL and the Cochrane Library. We included the most recently published high quality systematic reviews and any randomised controlled trials, non-randomised controlled trials, controlled before after studies or interrupted time series studies published subsequent to the systematic review. We searched for papers that evaluated pre-defined organisational interventions for adults with an ABI. Organisational interventions of interest included fee-for-service care, integrated care, integrated care pathways, continuity of care, consumer engagement in governance and quality monitoring interventions. Data extraction and appraisal of included reviews and studies was completed independently by two reviewers.

**Results:**

A total of five systematic reviews and 21 studies were included in the review; eight of the papers (31%) included people with a traumatic brain injury (TBI) or ABI and the remaining papers (69%) included only participants with a diagnosis of stroke. We found evidence supporting the use of integrated care to improve functional outcome and reduce length of stay and evidence supporting early supported discharge teams for reducing morbidity and mortality and reducing length of stay for stroke survivors. There was little evidence to support case management or the use of integrated care pathways for people with ABI. We found evidence that a quality monitoring intervention can lead to improvements in process outcomes in acute and rehabilitation settings. We were unable to find any studies meeting our inclusion criteria regarding fee-for-service care or engaging consumers in the governance of the health care organisation.

**Conclusions:**

The review found evidence to support integrated care, early supported discharge and quality monitoring interventions however, this evidence was based on studies conducted with people following stroke and may not be appropriate for all people with an ABI.

**Electronic supplementary material:**

The online version of this article (doi:10.1186/1472-6963-14-397) contains supplementary material, which is available to authorized users.

## Background

Acquired brain injury is the most common cause of disability worldwide [1–3]. The term ABI encompasses a number of different conditions including traumatic brain injury, hypoxic brain injury, stroke and brain tumour [4]. These conditions may cause a complex combination of symptoms that require treatment from multiple health professionals. The effects of an ABI may be long-lasting and result in the need for long-term management [5]. The incidence of ABI, frequency of limitations in functional ability that results from ABI, and costs associated with long term care mean that it is important that policy makers and health care organisations use the most effective and efficient methods to organise patient care.

Currently, the organisation of health care services for ABI varies across healthcare settings and countries and there is a lack of synthesised information regarding effective organisational interventions. Structuring services for people with an ABI is complex due to differences in case presentation, occupational goals and medical and functional needs. Differences between ABI aetiologies also lead to variation; the effects of stroke on the brain are much more focal in nature whereas TBI tends to lead to more generalised damage [6]. The needs of patients with ABI are often diverse [5], for example patients with stroke are more likely to be older, retired and may have made previous adjustments to their lifestyle due to ageing, whereas patients with traumatic brain injury tend to be of working age and are more likely to have family and work commitments. That said, the number of elderly people with TBI is on the rise and these patients tend to have worse medical outcomes than elderly stroke patients [7]. Despite differences in characteristics and treatment goals, people with an ABI from all diagnoses are often treated in the same care settings and by the same health care team and indeed, current research suggests that care on a mixed neurological rehabilitation unit is not inferior to care in a diagnostic-specific rehabilitation unit [8]. Understanding the research underpinning care provision across ABI is important because optimising care coordination can maximise rehabilitation potential, therefore optimising independence and quality of life.

One such source of evidence available to guide clinical care is the Evidence Based Review of Moderate to Severe Acquired Brain Injury (ABIEBR) ( https://www.abiebr.com) [9]. The ABIEBR is a continually updated review of evidence based interventions for persons with ABI. The ABIEBR includes several recommendations related to the organisation of care including: that care should be provided by dedicated multidisciplinary teams; with adherence to acute care guidelines, that rehabilitation should be provided early and at high intensity; and patients should have access to long term support and specialist programs (such as vocational rehabilitation). These recommendations were based on studies identified in their search; many of these were of low quality and thus the authors conclude that there is currently “insufficient evidence to draw any conclusions regarding the ideal structure of a complete model of ABI care” (p55). The definition of ABI used in the ABIEBR does not include stroke and thus, the large amount of research related to stroke is not considered in their summaries to date. The objective of this review was to identify the most up-to-date, high quality sources of evidence to answer specific questions of interest regarding organising health services for people with an acquired brain injury, inclusive of stroke.

## Methods

This systematic review was undertaken to inform the development of a new brain injury rehabilitation unit. The research team were therefore interested in organisational interventions, that is, interventions that relate to the structure or delivery of health services. The research team identified seventeen relevant systematic review questions from the Cochrane Effective Practice and Organisation of Care (EPOC) Group taxonomy of interventions. The questions were presented to key stakeholders in the brain injury rehabilitation unit and the key stakeholders voted for the questions that were perceived to be of highest priority. Key stakeholders included ABI clinicians, healthcare administrators and researchers. Six research questions related to the organisation of health care services were addressed within this systematic review (see list of Research questions). A protocol for the review was developed and registered with PROSPERO prior to undertaking the search [10]. As the review involved the synthesis of already published research, ethics approval was not required.

### Inclusion criteria

#### Types of studies

The first step in the search was to identify high quality published systematic reviews for each question. Systematic reviews were not excluded on the basis of the types of primary studies included. Where there were more than one systematic review we included the most recent high quality review (as assessed using the AMSTAR checklist). We also included primary studies that were published subsequently to systematic reviews to ensure that we included the most recent evidence (see PRISMA flow diagram). Studies were considered if they were randomised controlled trials (RCTs), non-randomised controlled trials (NRCTs), controlled before after studies (CBAs) or interrupted time series studies (ITSs). Where we were unable to identify any systematic reviews to address a research question we summarised studies of the aforementioned designs. Studies published prior to 1980 were not included; the cut-off date was chosen in order to ensure consistency with the ABIEBR. Conference proceedings were not included. Details of SRs and studies omitted from this review and the reasons for omission are presented in an Additional file 1 (see file).

#### Types of participants

Participants in included reviews and studies were aged ≥ 16 years with an acquired brain injury (as a result of trauma, lack of oxygen to the brain, stroke, tumours, infection, poisoning or substance abuse) [4] utilising acute care or rehabilitation services.

#### Types of interventions

We included interventions that evaluated: (1) fee-for-service versus no fee-for-service or partial fee-for-service, (2) formal integration of services versus non-integrated care. Integrated care was defined as care provided by the same multidisciplinary team (this may have involved any configuration of medical, nursing or allied health) in comparison to care provided by two or more teams of any configuration. The multidisciplinary team providing the seamless care must have had the same direct management however may not have involved the same personnel) (3) care based on integrated care pathways versus usual care. An integrated care pathway (ICP) defines the expected course of events in the care of patients with a particular condition within a set time frame; ICPs are documented and staff are expected to adhere to the ICP. In order to be considered in this review, the pathway needed to be multidisciplinary (direct expected care behaviours in two or more professional groups). (4) a program of continuity of care versus no follow up, usual care or a lower quality model of continuity of care (eg fewer contacts involved). Studies examining the effect of follow up or case management within the same organisation or referral to one or more other organisations were included. The follow up was for *any* health care service (rehabilitation, counselling, or medication review) and was arranged following discharge from acute care or rehabilitation services. Case management was defined as the coordination of multidisciplinary care and reconciling this with patient needs. To be classified as case management, services needed to involve three or more of the following processes: entry screening, assessment, planning, coordination, monitoring and review, exit/closure planning, (5) consumer participation in governance versus no consumer participation or an alternative model of consumer participation, and (6) presence of quality monitoring systems versus no quality monitoring system or an alternative system.

#### Types of outcome measures

The primary outcome measure was patient outcome. This included outcomes measured at the level of activity or participation, health related quality of life, or mortality. The secondary outcomes were resource use, quality of care outcomes (such as adherence to recommended care) and participant satisfaction. Reviews or studies were not excluded based on the outcomes assessed.

The research questions addressed within the review are presented below:

### Research questions

1. Does the presence of a ‘fee-for-service’, paid either by the individual or insurer, improve outcomes for patients with ABI or the organisation in comparison to those where the individual or insurer does not pay ‘fee-for-service’?
2. Does an integrated care model where acute and rehabilitation, or admitted and community/ambulatory services are provided under one management team improve outcomes for patients with ABI or the organisation compared with care provided by separate management teams?
3. Does the use of an integrated care pathway (ICP) result in improved outcomes for the patient or the organisation compared to care which does not routinely adhere to an ICP?
4. Does enhancing the continuity of care by providing or organising follow up or case management improve outcomes for patients with ABI or the organisation in comparison to care where these services are not provided?
5. Does engaging consumers in governance of the health care organisation improve outcomes for patients with ABI or the organisation in comparison to models where there is no consumer participation in governance?
6. Does the presence or organisation of quality monitoring systems improve outcomes for patients with ABI or the organisation compared with those lacking quality monitoring systems?

### Searches undertaken

The following electronic databases were searched for eligible reviews and studies: Medline (Ovid) 1980-Week 4 January 2013; PsycINFO (Ovid) 1980-Week 4 January 2013; EMBASE (Ovid) 1980-Week 4 January 2013 and CINAHL (Ebsco) 1980-21^st^ February 2013. The search strategy was developed (by KL in consultation with all authors) for use in Ovid and adapted for CINAHL (see Additional file 2 MEDLINE search strategy). The Cochrane Library (Cochrane Database of Systematic Reviews, Database of Abstracts of Reviews and Effects and the Cochrane Central Register of Controlled Trials) was searched on the 26^th^ of January 2013 using the MESH terms ‘stroke’ and craniocerebral trauma’. The Cochrane EPOC Group trials register was searched on the 13^th^ of February 2013.

### Data collection and analysis

One author (KL) reviewed all titles and abstracts elicited in the search to determine whether they met the inclusion criteria. Papers identified as being potentially relevant were obtained in full text and reviewed independently by two people (KL and LP). Disagreements were resolved by discussion or moderation by a third author (NL). Reasons for the exclusion of studies obtained in full text were recorded. The following data was extracted by one author (KL) and checked for accuracy by a second person (LP): review or study authors, date of publication, setting, study design, participant eligibility criteria, number of participants, intervention details, comparator details, type and timing of outcome measures, results and the authors’ conclusion. Studies were assessed independently by two people (KL and LP or DW) using the AMSTAR checklist [11] for systematic reviews, PEDro scale [12] for RCTs or Downs and Blacks Scale [13] for all other study designs.

The details of the included papers were summarised by research question and study type and presented in tables. No additional quantitative synthesis was conducted. Levels of evidence were described using the classification system used within the ABIEBR [9] which was based on the levels of evidence used by the United Stated Agency for Health Care Policy and Research Guidelines for Stroke Rehabilitation. Within this classification system, evidence is described as Level I if the findings are supported by the results of one or more RCTs of at least good quality (PEDro ≥ 6) and Level II if the findings are supported by a single RCT of at least “fair quality” (RCT <6 PEDro), NRCTs and Cohort studies.

## Results

**Figure 1:**
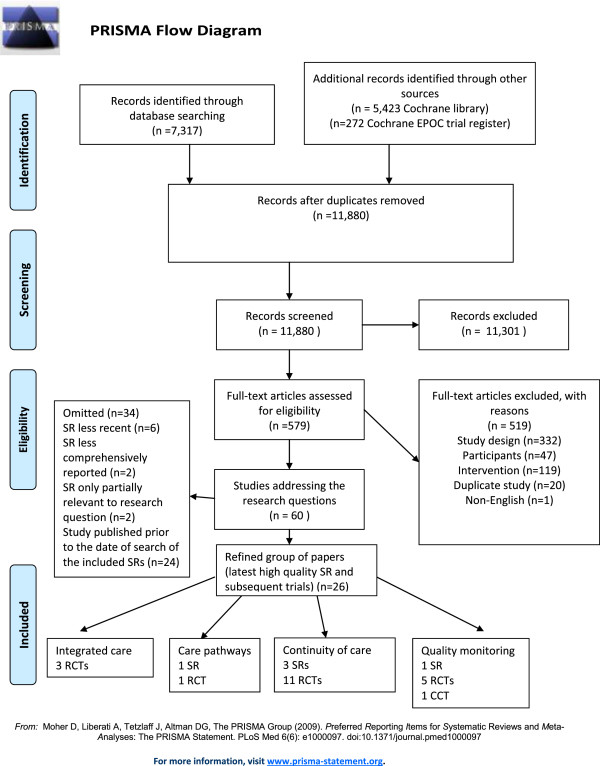
**PRISMA flow diagram.**

The search resulted in 11,880 citations of which 11,301 were excluded because the abstract revealed the study did not meet the inclusion criteria. Full text articles were obtained for the remaining 579 citations. A further 519 studies were excluded because they did not meet the inclusion criteria. We identified a total of 15 SRs, 43 RCTs and two NRCTs that addressed the study design, types of participants and intervention relevant to this review. We then reviewed the papers to identify the most recent, high quality systematic reviews and subsequently published studies; we included a total of five SRs, 20 RCTs and one NRCT in this final review. (Refer to Figure 1 PRISMA flow diagram).

We were unable to identify any SRs or studies meeting our inclusion criteria that addressed the research questions relating to fee-for-service or consumer involvement. Results for the remaining research questions are summarised below.

### Integrated care

#### Description of reviews and studies

We were unable to identify any SRs that examined the effectiveness of integrated care (where acute and rehabilitation, or admitted and community/ambulatory services were provided under one management team) compared to care provided by different management teams for people with an ABI. Three RCTs [14–16] were identified as meeting the inclusion criteria for this research question (See Table 1). No NRCT, CBA or ITS studies were identified which addressed this research question.

**Table 1:** **Integrated care**

Study	Patient	Intervention/Comparator	Outcomes	Results
**Indredavik 2000**	Patients with acute stroke	I: Chain of care provided by a mobile stroke team from the acute setting into the community	ADL function and independence, living situation, death, LOS	Patients receiving integrated care had improved functional outcome at 6 months (65% of the intervention group vs 52% of the control group were independent) and 1 year (56% of the intervention group vs 45% of the control group were independent). Length of stay and levels of mortality on the acute stroke unit were similar.
**Design: RCT**				
**Size: N = 320**				
**Setting: Hospital to home**				
**Norway**		C: Acute care and community care provided by different intervention teams		
**PEDro: 6/10**				
**Kalra 2000**	Patients with acute stroke	I: Comprehensive stroke ward (acute and rehabilitation care provided)	Mortality, ADL function, living situation, length of stay and resource use	There was a statistically significant reduction in mortality in the comprehensive care group in comparison to the group receiving general ward care (OR 0.39 (95% CI 0.20 to 0.76 at 6 months).
**Design: RCT**				
**Size: N = 457**				
**Setting: Hospital**		I: Domiciliary multidisciplinary stroke team		
**United Kingdom**				
**PEDro: 8/10**				
		C: General ward with care from hospital based mobile stroke team		
**Widen Holmqvist 1998**	Patients with acute stroke	I: Care provided by a hospital outreach team from the acute setting into the community	Resource use, caregivers QOL and time spent caring, patient satisfaction, Sickness Impact Profile, ADL function and independence	There were no statistically significant differences in outcome between groups at 3 or 12 months. Patients in the control group spent significantly more days in inpatient services (mean of 29 days vs mean of 14 days)
**Design: RCT**				
**Size: N = 81**				
**Setting: Hospital to home**		C: Acute care, rehabilitation and/or community care provided by different teams		
**Sweden**				
**PEDro: 7/10**				

One RCT conducted in Norway (n = 320) compared a ‘chain of care’ provided by one team from the acute stroke unit to the community with a more fragmented care approach [14]. The ‘chain’ started in the acute stroke unit, involved facilitation of discharge and coordination of additional rehabilitation and follow up in the community. The second trial (n = 81) also compared integrated care from the acute stroke unit to the home with usual care which involved at least two different health care teams [15]. The team providing the care were associated with the stroke unit and provided outreach rehabilitation in the home. Finally, a three-armed trial compared comprehensive stroke unit care which comprised acute care and rehabilitation to acute care provided by a mobile stroke team on general wards to care provided by a domiciliary stroke team [16]. There were several differences between the interventions provided in each of the study arms within this trial and the level of integration of care was one of several elements that varied. Consistent across all of the included studies (n = 3), was that they all recruited stroke survivors who predominantly had a moderate level of disability.

#### Quality of reviews and studies

The three included RCTs were all conducted in Europe and assessed as being of good quality (PEDro score ≥6/10) with large sample sizes (n = 81 to n = 320).

#### Effects of interventions

##### Patient outcomes

Two of the RCTs reported on mortality; of these, one of the trials found that there were reduced odds of mortality in the integrated care group at three, six and 12 months (OR 0.37; 95%CI 0.21 to 0.66 at 12 months) [16] whereas the other RCT found that there were similar levels of mortality between groups [14]. All three included RCTs reported on participants’ level of dependence in activities of daily living following intervention [15–17]. While one of the RCTs found no significant differences between groups [15], the others both reported more favourable outcomes for the integrated care group; Indredavik and colleagues (2000) reported that a higher proportion of participants were independent six months after stroke as measured using the Rankin Scale (65% vs 52%, odds ratio (OR) = 1.72; 95% CI 1.10 to 2.70) [14]. In addition, Kalra and colleagues found that more participants in the integrated care group had a favourable outcome on the Barthel Index (scores 15–20 out of 20) than those receiving care from the mobile stroke team at three (OR 1.16; 95% CI 1.02 to 1.32) and 12 months (OR 1.27; 95% CI 1.12 to 1.44) [16]. Two of the RCTs reported on the impact on health related quality of life. One of the RCTs found no significant differences between groups [15] whereas the other RCT found that participants in the intervention group reported significantly improved quality of life (mean score 78.9 vs 75.2) [14].

##### Secondary outcomes

All three RCTs reported on length of stay with variability in the findings. One RCT found that the total duration of hospital stay was similar between groups [16] while the remaining studies both reported a positive effect of the intervention on length of hospital stay. One RCT found that there was a 52% reduction in length of hospital stay for the integrated care group (mean of 14 days vs 29 days) [15] and the other found that the combined stroke unit plus rehabilitation unit length of stay was statistically shorter in the integrated care group (mean of 19 days vs 31 days) [14]. Finally, one of the RCTs assessed participant satisfaction and found that the group receiving integrated care reported statistically significant higher satisfaction with active participation in treatment program planning [15].

In summary, there is Level I evidence from three RCTs that the provision of integrated care may result in similar or reduced levels of mortality and similar or improved functional outcome when compared with a more fragmented approach. Furthermore, integrated care models used between the acute and home rehabilitation setting may result in a significantly reduced length of stay in acute services.

### Care pathways

#### Description of reviews and studies

We identified a Cochrane Review published in 2004 [17] and a RCT published in 2011 [18] that assessed the effects of integrated care pathways (ICPs) in stroke care (See Table 2). No NRCT, CBA or ITS studies were identified which addressed this research question. The Cochrane Review conducted by Kwan and colleagues included ten studies (three RCTs, seven NRCTs) with a total of 2013 participants. Care pathways were defined as a plan of care that “involved two or more of the following aspects of care: assessment, investigation, diagnosis, or treatment and involved two or more disciplines” [17]. The care provided for the control group was poorly described in all studies although appeared to be usual care and studies took place in both acute care and rehabilitation units. The included RCT conducted by Middleton and colleagues used a cluster design to evaluate the use of an ICP designed to manage fever, hyperglycaemia and swallowing on an acute stroke unit [18]. The trialists randomised 19 Australian acute stroke units and obtained data for 1696 participants.

**Table 2:** **Care pathways**

Study	Patient	Intervention/Comparator	Outcomes	Results
**Kwan 2004**	Patients with acute stroke	I: Integrated care pathway	Death or dependency, complications, readmission, use of investigations, patient satisfaction, LOS, cost of hospitalisation, QOL	There do not appear to be benefits in the implementation of an ICP and it is possible that use is associated with reduced patient satisfaction and QOL
**Design: SR**		C: No integrated care pathway		
**Size: 10 studies**				
**Setting: Acute**				
**AMSTAR: 7/11**				
**Middleton 2011**	Patients with stroke	I: Integrated care pathway directed at fever, hyperglycaemia and swallowing	Death or dependency, ADL function, QOL, LOS, processes of care	Patients managed using the ICP were less likely to be dead or dependent at 90 days (42% in the intervention group vs 58% in the control group, number needed to treat 6.4)
**Design: Cluster RCT**		C: No integrated care pathway		
**Size: N = 735**				
**Setting: Acute**				
**Australia**				
**PEDro: 9/10**				

#### Quality of reviews and studies

The Cochrane Review was of high quality meeting 7 of the 11 AMSTAR criteria however the authors reported that care needed to be taken in interpretation of the results as there were issues with the methodology and reporting of included studies.

The RCT conducted by Middleton and colleagues was of high quality (PEDro score = 9/10).

#### Effects of interventions

##### Patient outcomes

The SR and RCT both reported mortality rates post-intervention. The SR was unable to find any effects on mortality associated with use of an ICP. In contrast, the RCT found that patients receiving the intervention were significantly less likely to be dead or dependent at 90 days than patients receiving usual care (42% vs 58%, difference in absolute change = 15.7% (95% CI 5.8 to 25.4)). The SR and RCT both examined effects on quality of life; the SR identified evidence (based on one RCT [19]) that self-reported quality of life was significantly lower in the ICP group at six months whereas the RCT found that patients in the ICP group were more likely to have higher scores on the physical health component of the quality of life assessment tool (difference in absolute change = 3.4 (95% CI 1.2 to 5.5)) [19].

##### Secondary outcomes

The SR and RCT both reported on resource use; the SR reported that readmissions to hospital were lower in the ICP group (based on one RCT and one NRCT (OR 0.11, 95% CI 0.03 to 0.39). The data from the SR related to length of stay was conflicting with two RCTs [19, 20] finding increased length of stay and two NRCTs [21, 22] finding shorter length of stay in the ICP group. The RCT conducted by Middleton and colleagues found no significant differences in length of hospital stay between groups [19]. The SR examined the effects of ICP use on patient satisfaction and found some evidence (based on one randomised study [20] that patient satisfaction (as rated on a scale of one to ten) was significantly lower in participants receiving care based on an ICP (Weighted Mean Difference (WMD) -1.1, 95% CI −1.91 to −0.29).

Based on the available evidence from one SR, there is currently a lack of evidence supporting the implementation of ICPs to improve patient outcomes for people with ABI and limited evidence that ICPs may reduce patient satisfaction and quality of life compared to usual care. However, there is conflicting Level I evidence from one high quality RCT that a specific pathway used on an acute stroke unit to manage fever, hyperglycaemia and swallowing can improve patient outcome of death or dependency at 90 days compared with usual care [18].

### Continuity of care

A large number of reviews and studies were identified which addressed this research question. The purpose and content of intervention approaches varied and to assist in interpretation we categorised these into three main approaches: ‘continuity of care - case management’, ‘continuity of care - early supported discharge’ and ‘continuity of care - short term program based on consultation’. The ‘short term program’ category involved interventions that were short (one to three sessions), delivered by a health professional and focussed on assessment and subsequent referral to other agencies. In all categories interventions were conducted either face-to-face, via telephone calls or using a combination of these methods. Details of the individual studies are presented in Table 3 and findings are summarised by intervention category.

**Table 3:** **Continuity of care**

Study	Patient	Intervention/Comparator	Outcomes	Results
**Case management**				
**Ellis 2010**	Patients with stroke	I: Stroke liason worker	Subjective health, function, participation, death, institutionalisation, mood, stroke related knowledge, health service utilisation, patient satisfaction	Patients with mild to moderate disability had a significant reduction in dependence (OR 0.62, 95% CI 0.44 to 0.87) and there were reports of higher patient and carer satisfaction however there was no other evidence that the intervention improves outcome.
**Design: SR**		C: Alternative care or no post-discharge care		
**Size: 16 studies**				
**Setting: Predominantly community**				
**AMSTAR: 8/11**				
**Chesnut 1999**	Patients with TBI	I: Long term care coordination	General functional status	There was a lack of high quality studies and studies included in the review reported conflicting results therefore the authors were unable to make clear recommendations on the evidence for this approach.
**Design: SR**				
**Size: 3 studies**				
**Setting:**				
**Community**				
**AMSTAR: 3/11**				
**Allen 2009**	Patients with stroke	I: 6 months of follow up contact from an Advanced Practice nurse who worked with the GP to implement a care plan, organised further services and provided education	NIHSS, TUG, physical performance test, mortality, institutionalisation, QOL, mgt of post-stroke complications, stroke knowledge and lifestyle modification	There was little difference between groups at 6 months. The intervention group had slightly improved lifestyle management and stroke knowledge.
**Design: RCT**				
**Size: N = 380**				
**Setting: Acute to community**				
**United States**				
**PEDro: 8/10**		C: Usual care plus provision of written stroke related education.		
**Bell 2005**	Patients with TBI	I: Regular phone calls over 9 months upon discharge to follow-up any issues, identify concerns and provide information, mentoring, goal setting, reassurance and referral to community resources.	Function, community integration, neurobehavioural functioning inventory, Glasgow outcome scale, QOL, emotional state	The intervention group had significantly better scores on functional status and perceived quality of wellbeing than the control group however the magnitude of the effect is unclear as the outcome was a composite measure.
**Design: RCT**				
**Size: N = 171**				
**Setting: Rehabilitation to community**				
**United States**				
**PEDro: 8/10**		C: Usual care		
**Bell 2008**	Patients with mild TBI	I: Regular phone calls over 3 months post injury after presentation to the ED, a contact phone number and additional information about brain injury and where to get help	Head injury symptoms, QOL. PHQ, role performance, community participation	Patients in the intervention group reported fewer symptoms 6 months post injury than the control group (6.6 difference in adjusted mean symptom score, 95% CI 2.2 to 5.2)
**Design: RCT**				
**Size: N = 366**				
**Setting: ED to community**				
**United States**				
**PEDro: 8/10**				
		C: Usual care (patient handout and outpatient treatment if prescribed).		
**Bell 2011**	Patients with TBI	I: Regular phone calls for up to 21 months post injury from a case manager. The purpose of the calls was to help participants to identify, prioritise and solve problems as independently as possible	Function, level of disability, participation, symptoms, QOL, vocational status	No significant differences were found between groups for any of the measures at either 1 or 2 years post injury.
**Design: RCT**				
**Size: N = 433**				
**Setting: Rehabilitation to community**				
**United States**				
**PEDro: 6/10**		C: Usual care		
**Trexler 2010**	Patients with ABI	I: Allocated to ‘resource facilitators’ who contacted participants every 2 weeks (via telephone or home/community visits). A large focus of the facilitator was returning the patient to work (CM)	Participation, self reported health	Levels of participation improved more in the intervention group (*F* = 9.11) and more of the intervention group were employed at the time of 6 month follow up (n = 7 vs n = 4).
**Design: RCT**				
**Size: N = 22**				
**Setting: Community**				
**United States**				
**PEDro: 4/10**		C: Usual care (no ‘resource facilitator’		
**Early supported discharge**				
**Fearon 2012**	Patients with acute stroke	I: Early supported discharge	Death or long term dependency, length of stay, ADL function, subjective health status, mood, carer outcome, patient and carer satisfaction	Appropriately resourced Early Supported Discharge models can reduce length of stay (equivalent to approximately 7 days). Patients receiving ESD are more likely to be independent and living at home OR 0.80 (95% CI 0.67 to 0.97)
**Design: SR**		C: Other models of care		
**Size: 14 studies**				
**Setting: Acute stroke to home**				
**AMSTAR: 9/11**				
**Short term package**				
**Andersen 2000**	Patients with stroke	Ia: Three home visits from a geriatric rehabilitation physician for medication review, referral/liason with other services, information	Function, ADLs, mortality, institutionalisation and readmission	Significantly less hospital readmissions in both intervention groups compared to the control group at 6 months (26% vs 34% and 44%).
There was no difference in functional outcome between groups at 6 months.				
**Design: RCT (three arms)**				
**Size: N = 155**				
**Setting: Rehabilitation to community**		Ib: Home visits (average of 3) from the hospital physiotherapists for instruction and education		
**Denmark**				
**PEDro: 7/10**				
		C: Standard aftercare (may have included outpatient rehabilitation and home care)		
**Forster 2009**	Patients with stroke	I: Follow up assessment from a stroke nurse 5–6 months after stroke. Issues identified in the assessment were managed in a standardised manner	Function, mood, satisfaction with care, caregiver burden	There were no real differences between groups at 12 month follow up however patients reported improved satisfaction with care in some areas.
**Design: RCT**				
**Size: N = 265**				
**Setting:**				
**Community**				
**United Kingdom**		C: Letter sent to GP recommending 6 month review		
**PEDro: 8/10**				
**Ghaffar 2006**	Patients with mild TBI	I: Patients were followed up in a multidisciplinary TBI clinic within 1 week of injury and offered pharmacotherapy, PT, OT and supportive psychotherapy if required	Symptoms, General Health questionnaire, cognition	In general there were no significant differences between group however a small subgroup (those with a premorbid psychiatric history) appeared to benefit from treatment, reporting lower levels of depression at 6 months (*F* = 6.8).
**Design: RCT**				
**Size: N = 191**				
**Setting: ED to community**				
**Canada**				
**PEDro: 5/10**		C: Usual care (no follow up arranged)		
**Wade 1997**	Patients with head injury	I: Routine follow up from an occupational therapist or psychologist 7–10 days post injury with organisation of further follow up as required	Symptoms	As a whole, there were no significant differences between groups however subgroup analyses revealed that patients in the control group with a more severe head injury were more likely to have continuing problems at 6 months.
**Design: RCT**				
**Size: N = 1156**				
**Setting: ED to community**				
		C: Usual care (no routine followup)		
**United Kingdom**				
**PEDro: 3/10**				
**Wade 1998**	Patients with head injury	I: Routine follow up from an occupational therapist or psychologist 7–10 days post injury with organisation of further follow up as required	Symptoms	The intervention group reported fewer or less severe concussion symptoms and less disruption of social activities at 6 months than the control group.
**Design: RCT**				
**Size: N = 314**				
**Setting: ED, hospital and community**				
		C: Usual care (no routine followup)		
**United Kingdom**				
**PEDro: 5/10**				
**Ytterberg 2000**	Patients with stroke	I: All day follow up visit one month after discharge	Self reported health status	There were no significant differences between groups
**Design: RCT**				
		C: Usual care with no specific follow-up arranged		
**Size: N = 111**				
**Setting: Community**				
**Sweden**				
**PEDro: 2/10**				

#### Continuity of care - case management

##### Description of reviews and studies

In the category of case management we included two SRs due to differences in the participants involved; one involved only people with stroke and the other involved only people with TBI. A Cochrane review published by Ellis and colleagues in 2010 included 16 RCTs that examined the effectiveness of stroke liaison workers for patients following stroke [23]. We found one RCT (n = 380) published after the search date of the SR that examined the effects of a six month case management intervention for people with stroke on discharge from acute care [24]. In addition, a systematic review published in 1999 examined the effectiveness of case management for patients following traumatic brain injury [25]. Since then, three RCTs involving people with a TBI have assessed the evidence for a case management intervention [26–28]. Another RCT examined the evidence for case management focussed on returning people with an ABI to work [29]. The participant group comprised seven people with TBI, six people with cerebral infarcts, seven people with intracranial haemorrhage and two people with other diagnoses.

#### Quality of reviews and studies

The SR involving people with stroke was recently published, involved a large number of RCTs (n = 16) and addressed the majority of the AMSTAR criteria (8/11) [23]. In contrast, the SR involving people with TBI [25] was published in 1999 and addressed few of the AMSTAR criteria (3/11). Their review comprised three low quality studies, one of which was randomised. The RCT conducted with stroke survivors was of high quality (PEDro 8/10). The three RCTs conducted with people with a TBI were of good quality (PEDro ≥6/10) whereas the RCT conducted with people with an ABI was of poorer quality (PEDro 4/10) and involved a small number of participants (n = 22).

#### Effects of interventions

##### Patient outcomes

The SR conducted with stroke survivors found that there were no differences between groups on mortality at follow up [23]. Both SRs [23, 25] and two of the RCTs [26, 28] reported on levels of dependence in ADL function post intervention. Both SRs found that there were no significant differences between groups. However, Ellis and colleagues found in a subgroup analysis that stroke survivors with mild to moderate disability (Barthel score of 15 to 19) who received case management were more likely to be have a significant reduction in dependence (OR 0.62, 95% CI 0.44 to 0.87) in comparison to those who did not receive case management. One RCT conducted with people with TBI found that patients receiving case management had improved functional status (on a composite outcome measure) at follow up however the magnitude of the effect was unclear [28], whereas another RCT involving people with TBI found there were no significant differences between groups in ADL function measured using the Functional Independence Measure and Disability Rating Scale at follow-up [26]. Both SRs and four of the RCTs reported on levels of participation at follow up. The SR involving stroke survivors found that there were no significant differences between groups at follow up. The SR involving people with a TBI reported that there was some evidence (based on two NRCTs) that case management improved vocational status. The three RCTs conducted with people with TBI showed conflicting results; two of the RCTs were unable to find differences between groups in participation outcomes [26, 28] whereas the other RCT found that people in the intervention group reported less impact on work and leisure roles than those in the control group at follow up [27]. The RCT involving people with an ABI provided a case management intervention focussed on returning people to work and found that at six month follow up a higher number of people from the intervention group were employed (64% vs 36%) [29]. One SR [23] and five RCTs [24, 26–29] examined the effects of case management intervention on quality of life. The SR and four of the RCTs found there were no differences in outcomes between groups [23, 24, 26, 27, 29]. The remaining study found that the intervention group had significantly better adjusted mean scores on the Euroqol (0.78 vs 0.67) [28].

##### Secondary outcomes

The SR involving stroke survivors reported on participant satisfaction [23]; they found evidence (based on three RCTs included in their review) suggesting that participants receiving case management were more satisfied that someone had listened to them (OR 1.58, 95% CI 1.14 to 2.19).

In summary, one systematic review containing 16 RCTs and one RCT published since the review suggest that there are few overall significant benefits in providing case management services for people with stroke however there may be some benefits in providing case management for people with mild to moderate levels of disability. In addition, case management may increase satisfaction with care. One systematic review and four RCTs provided inconclusive evidence regarding the effectiveness of case management for people with TBI.

#### Continuity of care - Early supported discharge (ESD)

##### Description of reviews and studies

We identified a Cochrane review published in 2012 that examined the effect of early supported discharge programs after stroke [30]. Early supported discharge was defined in the review as “any intervention that aimed to accelerate discharge from hospital with the provision of support (with or without a 'therapeutic' rehabilitation intervention) in a community setting”. The review included 14 RCTs. No RCT, NRCT, CBA or ITS studies were identified which were published subsequently to this review and addressed this research question.

#### Quality of reviews and studies

The review was well reported, addressing 9 out of 11 of the AMSTAR criteria.

#### Effects of interventions

##### Patient outcomes

The SR reported on level of dependence in ADL and found that based on the results of nine trials there were no significant differences between groups. However, the SR found that patients receiving an ESD program were marginally more independent in extended ADL (standardised mean difference (SMD) 0.12 (95% CI 0.00 to 0.25)). The SR found no significant effect on quality of life or mortality. However, when assessing the outcome of death or dependency they found strong evidence that ESD programs led to similar or improved functional outcome (OR 0.80 (95% CI 0.67 to 0.97)) [30]. Subgroup analysis showed that patients with mild to moderate levels of disability (initial Barthel index >9/20) appeared to benefit most from ESD intervention however, it should be noted that the benefits associated with ESD were not always sustained at longer term follow up assessment [30].

##### Secondary outcomes

The SR reported on the effect on length of stay and found ESD intervention was associated with a significant reduction in length of stay in acute care equivalent to approx seven days (Mean Difference (MD) -6.84 (95% CI −11.20 to −2.49)). Overall patients receiving ESD services were significantly more likely to report satisfaction with care services (OR 1.60, 95% CI 1.08 to 2.38, P = 0.02).

In summary, there is Level I evidence that ESD programs can improve functional outcomes, reduce length of stay and increase patient satisfaction. The authors of the review reached a consensus that the important factors of the ESD service included delivery of therapy in the home setting and coordinated multidisciplinary care. The greatest benefits were seen where the ESD team coordinated the hospital discharge, post-discharge care and delivery of home rehabilitation and support.

#### Continuity of care - Short term programs

##### Description of reviews and studies

We identified six RCTs in this category [31–36]; three of these studies involved people with a traumatic brain injury and the remaining involved people with stroke. No SRs, NRCT, CBA or ITS studies were identified which addressed this research question. The purpose of the intervention, and health professionals delivering the intervention in this category varied greatly. Study interventions included: visits from a geriatric rehabilitation physician or physiotherapist [31], assessment conducted by a stroke nurse six months after stroke [32], follow up in a multidisciplinary TBI clinic [33], routine follow up approximately one week post TBI from an occupational therapist or psychologist [34, 35] and follow up one month following discharge after stroke [36].

#### Quality of reviews and studies

As seen in Table 3, PEDro scores for included studies tended to be low and scores ranged from 2/10 to 8/10.

#### Effects of interventions

##### Patient outcomes

There were few significant findings in favour of short term programs of continuity of care. One RCT reported on mortality outcomes and found no significant differences between groups [31]. Two RCTs reported on level of dependence in ADL and found no significant differences between groups [31, 32]. Five RCTs reported on participation outcomes; three of these found no significant differences between groups [31–33]. The remaining two RCTs were conducted with people with TBI and found that people in the control group were significantly more likely to have difficulties in participation (as measured by the Rivermead Head Injury follow up Questionnaire) than those in the intervention group [34, 35]. Two RCTs examined effects on quality of life and were unable to identify significant benefits associated with intervention [33, 36].

##### Secondary outcomes

One of the RCTs assessed participant satisfaction and found that patients receiving the intervention reported that they were more satisfied with the information provided to them and the planning prior to their return home compared with usual care [31].

In summary, we identified a number of studies evaluating short term programs however, low study quality and clinical heterogeneity means that it is difficult to draw conclusions about these studies.

### Quality monitoring

#### Description of reviews and studies

We included one SR [37], five RCTs [20, 38–41] and one NRCT [42] that addressed this question (See Table 4). No CBA or ITS studies were identified which addressed this research question. The SR conducted by Parker and colleagues (2012) examined one aspect of quality monitoring; that is whether the evaluation of compliance with quality metrics or public reporting improved patient outcomes including mortality, disability, quality of life or patient satisfaction. They included 14 observational studies that examined compliance with quality metrics and two observational studies that examined public reporting; all studies took place in acute stroke settings. Four of the primary studies (3 RCTs, 1 NRCT) included in our review aimed to improve compliance with interventions regarded as best practice in acute care (for example, increasing the rates of thrombolysis to appropriate patients) [38–40, 42]. The quality monitoring intervention approaches used within these studies varied (Table 4). Two RCTs examined quality monitoring interventions in a stroke rehabilitation setting [20, 41]. The processes involved in the interventions were different however, the aim of both interventions was to improve team functioning and involved strategies such as audit, feedback and team training

**Table 4:** **Quality monitoring**

Study	Patient	Intervention/Comparator	Outcomes	Results
**Parker 2012**	Patients with stroke	I: Studies that evaluated the relationship between compliance with ≥ 2 quality metrics and patient centered outcomes or the public reporting of stroke metrics and QI activity, quality of care and patient centered outcomes.	Mortality, ADL function, adverse events/complications, QOL, patient satisfaction	There is some evidence of positive associations between stroke metric compliance and improved outcomes however, there are few high quality studies. Information on the impact of public reporting of stroke quality metric data is extremely limited
**Design: SR**				
**Size: 16 studies**				
**Setting: Acute**	All levels of severity			
**AMSTAR: 3/11**				
**Dirks 2012**	Patients with stroke	I: An intervention based on the ‘Breakthrough Series’ model to increase the rates of thrombolysis in acute stroke wards	Treatment rates of tPA, time from event to admission, death or disability, QOL	Thrombolysis rates in the intervention group rose earlier and remained higher than the control group.
		C: Usual care		
**Design: cluster RCT**				
**Size: N = 5515 patients from 12 hospitals**				
**Setting: Acute**				
**Netherlands**				
**PEDro: 7/10**				
**Falconer 1993**	Patients with stroke	I: Care was provided based on an interdisciplinary care model and the use of a ‘critical path method (CPM) to plan care and discharge. The CPM provided the team with information and continuous feedback	Length of hospital stay, hospital charges, ADL function, patient satisfaction	The groups received comparable type, intensity and duration of treatment and there was no significant difference between groups in length of stay and hospital charges
**Design: RCT**				
**Size: N = 128**				
**Setting: Rehabilitation**				
**United States**				
**PEDro: 4/10**				
		C: Usual care in which the care model was more multidisciplinary and a CPM was not used.		
**Hinchey 2010**	Patients with stroke	I: Multifaceted intervention targeted towards improving key performance measures: door-to-needle time for TPA, dysphagia screening, DVT prophylaxis and warfarin treatment for AF. The intervention included meetings, identification of barriers, reminder systems, education, audit and feedback.	Difference in post-intervention adherence rates	The intervention group had a significantly higher rate of patients with AF discharged on warfarin however there were no other significant differences between groups.
**Design: Controlled trial**				
**Size: N = 2071 pre-intervention patients and 1240 post-intervention patients**				
**Setting: Acute**				
**United States**		C: Audit and feedback alone		
**D & B: 13/26**				
**Johnston 2010**	Patients with stroke	I: Standardised stroke discharge orders on adherence to 3 practices: normalisation of blood pressure, statin treatment and anticoagulation for AF	Management of these outcomes at 6 months	There was no significant impact of intervention at the hospital level.
**Design: cluster RCT**				
**Size: 12 hospitals (3361 patients)**				
				Analysis at the patient level found that rates of optimal treatment increased at intervention hospitals whereas there was no change at control hospitals. Improvements were primarily related to increased statin use and improved blood pressure control.
**Setting: Acute**		C: Usual care (no standardised orders)		
**United States**				
**PEDro: 8/10**				
**Lakshminarayan 2010**	Patients with stroke	I:Intervention to improve care quality as measured by	Ten performance measures(eg tPA use, smoking cessation counselling, PT and OT evaluation or treatment <48 hours)	There were no significant differences between groups
**Design: cluster RCT**		10 key performance measures. Intervention included receipt of a report on baseline quality, the use of clinical opinion leaders and assistance from study personnel to implement changes and overcome barriers		
**Size: 19 hospitals (1211 patients)**				
**Setting: Acute**				
**United States**				
**PEDro: 8/10**				
		C: Received report on baseline quality only		
**Strasser 2008**	Patients with stroke	I: Both groups received summaries of their team’s performance on process measures. The intervention group received team training provided over 6 months. Comprised a 2.5 day workshop for team leaders to develop team problem-solving strategies, written action plans to address team process problems and support to implement action plans	ADL function, community discharge and length of stay	Patients in the intervention group improved significantly more on the FIM motor score than the control group (13.6% absolute difference in percentage of patients gaining more than 23 points)
**Design: cluster RCT**				
**Size: N = 487 patients**				
**Setting: Rehabilitation**				
**United States**				
**PEDro: 5/10**				
		C; Received the summary of performance only		

#### Quality of reviews and studies

The SR addressed 3 of the 11 AMSTAR criteria. The RCTs ranged in quality; two were of lower quality with PEDro scores of 4/10 [20] and 5/10 [41]. The remaining three RCTs were of good quality (≥6/10 on the PEDro scale) [38–40]. The NRCT scored 13/26 on the Downs and Black Scale reflecting methodological weaknesses [42].

#### Effects of interventions

##### Patient outcomes

The SR reported broadly on the effect on patient outcomes (which included mortality, disability, quality of life and patient satisfaction). They found conflicting evidence as approximately half of the sixteen studies included in the review found mostly positive relationships with patient outcome whereas the other half reported either limited or no significant relationship [37]. One good quality RCT reported on mortality following intervention and found no significant differences between groups [38]. Three RCTs reported on participants’ level of dependence in ADLs post intervention [20, 38, 41]. Findings were mixed; one of the RCTs found no significant difference [20] whereas another reported that patients in the intervention group made more improvement on the motor component of the Functional Independence Measure (13.6% absolute difference in percentage of patients gaining more than 23 points) [41]. In contrast, Dirks and colleagues found that although the quality monitoring intervention achieved their goal of increasing thrombolysis rates, patients in the intervention group were less likely to have a good clinical outcome (Modified Rankin Scale <3) at 3 months (adjusted OR, 0.56 (95% CI 0.42 to 0.74) [38]. This same RCT found there were no significant differences between groups in quality of life [37].

##### Secondary outcomes

Three RCTs reported on resource use. One RCT found that there were some cost savings associated with the intervention due to lower hospital admission costs (difference -$455 USD; 95% CI -$232 to -$679 USD) [38]. However, two RCTs conducted in stroke rehabilitation settings found no significant differences between groups in length of stay [20, 41]. Three RCTs [38–40] and one NRCT [42] reported on the effect of the quality monitoring intervention on process measures. The study conducted by Dirks and colleagues found that rates of thrombolysis were increased in the intervention group (44.3% vs 39.8% in the control group (difference 4.5%; 95% CI 3.1% to 5.9%) [38]. Johnston and colleagues found that standardised stroke discharge orders increased the rates of ‘optimal treatment’ in individual patients (from 37% to 45%) [39] and Hinchey and colleagues found significant improvements in achieving one of four targeted process outcomes (98% in the intervention group compared to 87% in the control group) which was discharging a patient with atrial fibrillation on warfarin. Only one of the studies was unable to find any significant benefits in favour of the intervention group on process outcomes [40]. One RCT reported on participant satisfaction and found that patients in the intervention group reported significantly lower levels of satisfaction with intervention (mean 7.7 vs 8.8) [20].

In summary, level I evidence from one systematic review and six clinical trials indicates that while some improvements in processes outcomes have been associated with quality monitoring interventions, the effects on patient and health system outcomes are not consistent. There is not clear evidence supporting a particular approach to quality monitoring, and the primary studies evaluating these interventions were of variable methodological quality.

## Discussion

This review addressed six research questions; we found eligible papers to address four of these six questions. We included five systematic reviews and 21 trials (20 of which were randomised) in this review to answer these four questions. The majority of studies involved people with stroke rather than TBI, and most of the studies involved participants with mild to moderate levels of disability rather than people with a severe ABI. The RCTs were of mixed quality; most (n = 13, 65%) were assessed as being good quality with a PEDro score of ≥6/10.

Overall we found some evidence from three RCTs supporting integrated care models, and strong evidence from one systematic review for the use of early supported discharge teams after stroke. We found there was little evidence (based on one systematic review and one RCT) to support case management services for people with stroke and there was insufficient evidence from which to draw conclusions regarding case management for people with TBI (based on one systematic review and four RCTs). There was little evidence from one systematic review and one RCT to support the use of integrated care pathways and while some quality monitoring interventions have lead to improvements in patient care, results are inconsistent and there is insufficient evidence to support one particular quality monitoring approach (based on one systematic review and six clinical trials).

This review is the first known review of its type in ABI and provides useful information for health care service providers, funding bodies or policy makers wishing to establish or modify existing services for people with an ABI. This review complements the ABIEBR [9] module on models of care by including studies involving people with stroke and thus synthesising a larger body of literature.

The interventions of interest within this review were complex interventions; this presents issues in interpretation of the research. For example, the studies examining the effect of integrated care compared different interventions in which the degree of integration was only one of the possible explanatory variables. The study conducted by Kalra and colleagues compared comprehensive stroke care provided in a discrete location with care provided by a mobile stroke team [16]. It is possible that benefits in the comprehensive care group were related to other factors such as the dedicated nursing staff on the comprehensive unit.

Our finding regarding the lack of evidence for integrated care pathways warrants further discussion. The systematic review by Kwan and colleagues was published in 2002 and only identified three RCTs. The RCTs failed to identify any significant benefits following the introduction of ICPs and suggest that a “one size fits all” approach does not work. However, two of these RCTs [20, 43] were poorly reported and published in the early 1990s and as such, findings from these studies may be of limited relevance to current clinical practice. The more recent RCT conducted by Middleton and colleagues [18] was of high quality and much more specific in nature than the ICPs included within the 2002 systematic review. This type of pathway may be more effective and further high quality research is warranted to determine the effect of similar pathways for other impairments following ABI.

Evaluation of quality monitoring interventions is challenging in that the different quality monitoring interventions may vary in their effectiveness and their effectiveness may in turn vary in different contexts and settings. The baseline performance of the multidisciplinary team, receptiveness to intervention and the drivers of change in each setting may also influence the outcome, and should thus be reported in any trial. It is possible that clinicians may respond more positively to feedback regarding individual performance outcomes than team outcomes; this was also not addressed in any of the included studies. The majority of reviews and studies included within this review relate to people with mild to moderate levels of disability in terms of their level of independence in activities of daily living. The focus on people with less severe disability may partly relate to the nature of the research questions. Case management or follow up programs tend to be directed at people returning to their own home who are less severely disabled than those discharged to residential care services. This may also be due to the presence of less research related to people with higher levels of disability who may be excluded from studies due to communication or cognitive impairment. However, long term care for people with severe levels of disability is costly and people with severe levels of disability may gain more from interventions directed at improving their quality of life. More research is required to evaluate the best methods of organising health care services for this group. Most of the included studies also recruited people with stroke rather than other forms of ABI. As previously described, in most settings worldwide people with an ABI tend to be treated by the same team in the same settings; therefore researchers should consider a pragmatic approach by including all patients with ABI. While the needs and goals of people with different diagnoses may vary, there are areas of commonalities and the needs of all people with an ABI should be considered in the establishment of services.

There are some limitations associated with this review. Firstly, although we included research designs of the highest quality, the way in which some studies were reported suggests that they were susceptible to bias and therefore results should be interpreted with caution. All studies were appraised and results of the appraisal were presented in Tables 1, 2, 3 and 4. In addition, studies of particularly high or low quality were noted in the text. Our search for eligible studies was highly sensitive yielding a large volume of titles and abstracts; however it is possible that publication bias is present as we excluded conference proceedings and did not search clinical trial registries or contact authors. Furthermore, one author (KL) screened titles and abstracts identified from the search and one person (KL) extracted data (checked by a second author (LP)). We also did not include studies published prior to 1980 and those published in languages other than English.

We summarised the evidence using the same classification system as the ABIEBR to ensure consistency however, this was at the expense of using other more highly regarded systems such as GRADE [44]. There are many factors involved in designing or reviewing health care services. This review addresses specific organisational interventions but does not address issues such as the level of staffing and personnel that should be involved in providing care.

## Conclusions

Based on the evidence identified in the review we identified several implications for the establishment or review of the organisation of health care services for people with ABI. We have also identified areas in which there is conflicting evidence and where more evidence is required. Our findings suggest: there is Level I evidence that improved outcomes, including a shorter length of stay, are possible under an integrated care model where acute, rehabilitation, and community/ambulatory services are provided under one management team; there is Level I evidence that compliance with a specific integrated care pathway in the acute setting may result in improved patient outcomes and reduced mortality in stroke patients; case management after inpatient rehabilitation for stroke patients is associated with few reported benefits for stroke survivors; case management for patients with TBI has not yet been well studied; and there is conflicting evidence that quality monitoring interventions may lead to improved patient outcomes however at present, no particular quality monitoring approach can be recommended.

## Electronic supplementary material

Additional file 1: **SRs and studies omitted in the refinement phase.** (DOC 40 KB)

Additional file 2: **MEDLINE search strategy.** (DOC 28 KB)
