# Supplementary material for: Organising health care services for people with an acquired brain injury: an overview of systematic reviews and randomised controlled trials
Source: BMC Health Serv Res. 2014 Sep 17;14:397. doi: 10.1186/1472-6963-14-397 (PMC4263199; doi:10.1186/1472-6963-14-397)
Supplement: Supplementary file 1 — Additional file 1: SRs and studies omitted in the refinement phase. (DOC 40 KB) [file 12913_2014_3523_MOESM1_ESM.doc]

Additional file: SRs and studies omitted in the refinement phase

Integrated Care

1. Stroke Unit Trialists Collaboration: **Organised inpatient (stroke unit) care for stroke**. *Cochrane Database of Systematic Reviews* 2007(4):CD000197.

SR omitted as it only partially addressed our research question. The primary objective was to compare stroke unit care with alternative forms of care. Within this objective the authors searched for evidence regarding the effects of comprehensive stroke ward care versus alternative forms of care.

2. Fearon P, Langhorne P: **Services for reducing duration of hospital care for acute stroke patients**. *Cochrane Database of Systematic Reviews* 2012, **9**.

SR omitted from this section as it only partially adressed intgrated care. The primary aim of the review was to compare Early Supported Discharge services with other models of care.

Integrated Care Pathways

3. Sulch D, Kalra L: **Integrated care pathways in stroke management**. *Age & Ageing* 2000, **29**(4):349-352.

SR addressed the research question however was omitted as not as current as the included SR

4. Sulch D, Perez I, Melbourn A, Kalra L: **Randomized controlled trial of Integrated (managed) Care Pathway for stroke rehabilitation**. *Stroke* 2000, **31**(8):1929-1934

RCT omitted as published prior to the search date of the included SR

Continuity of Care

*Case Management*

5. Allison R, Shelling L, Dennett R, Ayers T, Evans PH, Campbell JL: **The effectiveness of various models of primary care-based follow-up after stroke: a systematic review**. *Primary Health Care Research & Development* 2011, **12**(3):214-222.

SR omitted as met fewer of the AMSTAR criteria (AMSTAR 5/11)

6. Graven C, Brock K, Hill KD, Joubert L: **Are rehabilitation and/or care coordination interventions delivered in the community effective in reducing depress, faciliation participation and improving quality of life after stroke?** *Disability & Rehabilitation* 2011, **33**(17-18):1501-1520.

SR omitted as met fewer of the PRISMA criteria (AMSTAR 4/11)

7. Salter K, Foley N, Teasell R: **Social support interventions and mood status post stroke: a review**. *International Journal of Nursing Studies* 2010, **47**(5):616-625.

SR addressed the research question however eas omitted as not as current as the included SR

8. Boter H: **Multicenter randomized controlled trial of an outreach nursing support program for recently discharged stroke patients**. *Stroke* 2004, **35**(12):2867-2872.

RCT omitted as published prior to the search date of the included SR

9. Christie D, Weigall D: **Social work effectiveness in two-year stroke survivors: a randomised controlled trial**. *Community Health Studies* 1984, **8**(1):26-32.

RCT omitted as published prior to the search date of the included SR

10. Claiborne N: **Effectiveness of a care coordination model for stroke survivors: A randomized study**. *Health and Social Work* 2006, **31**(2):87-96.

RCT omitted as published prior to the search date of the included SR

11. Forster A, Young J: **Specialist nurse support for patients with stroke in the community: a randomised controlled trial [see comments]**. *BMJ* 1996, **312**(7047):1642-1646.

RCT omitted as published prior to the search date of the included SR

12. Goldberg G, Segal ME, Berk SN, Schall RR, Gershkoff AM: **Stroke transition after inpatient rehabilitation**. *Topics in Stroke Rehabilitation* 1997, **4**(1):64-79.

RCT omitted as published prior to the search date of the included SR

13. Greenwood RJ, McMillan TM, Brooks DN, Dunn G, Brock D, Dinsdale S, Murphy LD, Price JR: **Effects of case management after severe head injury [see comments]**. *BMJ* 1994, **308**(6938):1199-1205.

RCT omitted as published prior to the search date of the included SR

14. Joubert J: **Risk factor management and depression post-stroke: the value of an integrated model of care**. *Journal of clinical neuroscience* 2006, **13**:84-90.

RCT omitted as published prior to the search date of the included SR

15. Lincoln N, Francis VM, Lilley SA, Sharma A, Summerfield M: **Evaluation of a stroke family support organiser: a randomized controlled trial**. *Stroke* 2003, **34**(1):116-121.

RCT omitted as published prior to the search date of the included SR

16. Mant J, Carter J, Wade D, Winner S: **Family support for stroke: a randomised controlled trial**. *Lancet* 2000, **356**:808-813.

RCT omitted as published prior to the search date of the included SR

17. Mayo NE, Nadeau L, Ahmed S, White C, Grad R, Huang A, Yaffe MJ, Wood-Dauphinee S: **Bridging the gap: the effectiveness of teaming a stroke coordinator with patient's personal physician on the outcome of stroke**. *Age and ageing* 2008, **37**(1):32-38.

RCT omitted as published prior to the search date of the included SR

18. Tilling K, Coshall C, McKevitt C, Daneski K, Wolfe C: **A family support organiser for stroke patients and their carers: a randomised controlled trial**. *Cerebrovascular Diseases* 2005, **20**(2):85-91.

RCT omitted as published prior to the search date of the included SR

*Early Supported Discharge*

19. Larsen T, Olsen TS, Sorensen J: **Early home-supported discharge of stroke patients: a health technology assessment**. *International Journal of Technology Assessment in Health Care* 2006, **22**(3):313-320.

SR addressed the research question however eas omitted as not as current as the included SR

20. Outpatient service trialists: **Therapy based rehabilitation services for stroke patients at home**. *Cochrane Database of Systematic Reviews* 2003, **1**.

SR addressed the research question however eas omitted as not as current as the included SR

21. Teasell R, Foley N, Bhogal S, Speechley M: **Early supported discharge in stroke rehabilitation**. *Topics in Stroke Rehabilitation* 2003, **10**(2):19-33.

SR addressed the research question however eas omitted as not as current as the included SR

22. Winkel A, Ekdahl C, Gard G: **Early discharge to therapy-based rehabilitation at home in patients with stroke: a systematic review** *Physical Therapy Reviews* 2008, **13**(3):167-187.

SR addressed the research question however eas omitted as not as current as the included SR

23. Askim T, Rohweder G, Lydersen S, Indredavik B: **Evaluation of an extended stroke unit service with early supported discharge for patients living in a rural community. A randomized controlled trial**. *Clin Rehabil* 2004, **18**(3):238-248.

RCT omitted as published prior to the search date of the included SR

24. bautz-Holter E, Sveen U, Rygh J, Rodgers H, Wyller TB: **Early supported discharge of patients with acute stroke: a randomized controlled trial**. *Disability & Rehabilitation* 2002, **24**(7):348-355.

RCT omitted as published prior to the search date of the included SR

25. Indredavik B, Fjaertoft H, Ekeberg G, Loge A, Morch B: **Benefit of an extended stroke unit service with early supported discharge: A randomized, controlled trial**. *Stroke* 2000, **31**(12):2989-2994.

RCT omitted as published prior to the search date of the included SR

26. Gilbertson L, Langhorne P, Walker AM, Allen A, Murray G: **Domiciliary occupational therapy for patients with stroke discharged from hospital: randomised controlled trial**. *BMJ (Clinical research ed)* 2000, **320**(7235):603-606.

RCT omitted as published prior to the search date of the included SR

27. Grasel E, Biehler J, Schmidt R, Schupp W: **Intensification of the transition between inpatient neurological rehabilitation and home care of stroke patients. Controlled clinical trial with follow-up assessment six months after discharge**. *Clinical Rehabilitation* 2005, **19**(7):725-736.

RCT omitted as published prior to the search date of the included SR

28. Logan PA, Ahern J, Gladman JR, Lincoln NB: **A randomized controlled trial of enhanced Social Service occupational therapy for stroke patients**. *Clinical Rehabilitation* 1997, **11**(2):107-113.

RCT omitted as published prior to the search date of the included SR

29. Markle-Reid M, Orridge C, Weir R, Browne G, Gafni A, Lewis M, Walsh M, Levy C, Daub S, Brien H *et al*: **Interprofessional stroke rehabilitation for stroke survivors using home care**. *The Canadian Journal of Neurological Sciences/ Le Journal Canadien Des Sciences Neurologiques* 2011, **38**(2):317-334.

RCT omitted as published prior to the search date of the included SR

30. Mayo N, Wood-Dauphinee S, Cote R, Gayton D, Carlton J, Buttery J, Tamblyn R: **There's no place like home : an evaluation of early supported discharge for stroke**. *Stroke* 2000, **31**(5):1016-1023.

RCT omitted as published prior to the search date of the included SR

31. Powell J, Heslin J, Greenwood R: **Community based rehabilitation after severe traumatic brain injury: a randomised controlled trial**. *Neurology, Neurosurgery and Psychiatry* 2002, **72**:193-202.

RCT omitted as published prior to the search date of the included SR

32. Rodgers H, Soutter J, Kaiser W, Pearson P, Dobson R, Skilbeck C, Bond J: **Early supported hospital discharge following acute stroke: pilot study results**. *Clinical Rehabilitation* 1997, **11**(4):280-287.

RCT omitted as published prior to the search date of the included SR

33. Rudd AG, Wolfe CD, Tilling K, Beech R: **Randomised controlled trial to evaluate early discharge scheme for patients with stroke**. *BMJ* 1997, **315**(7115):1039-1044.

RCT omitted as published prior to the search date of the included SR

34. Torp CR, Vinkler S, Pedersen KD, Hansen FR, Jorgensen T, Willaing I, Olsen J: **Model of hospital-supported discharge after stroke**. *Stroke; a journal of cerebral circulation* 2006, **37**(6):1514-1520.

RCT omitted as published prior to the search date of the included SR
