# Supplementary material for: Organising health care services for people with an acquired brain injury: an overview of systematic reviews and randomised controlled trials
Source: BMC Health Serv Res. 2014 Sep 17;14:397. doi: 10.1186/1472-6963-14-397 (PMC4263199; doi:10.1186/1472-6963-14-397)
Supplement: Supplementary file 2 — Additional file 2: MEDLINE search strategy. (DOC 28 KB) [file 12913_2014_3523_MOESM2_ESM.doc]

MEDLINE search strategy

1 exp Craniocerebral Trauma/
2 exp Stroke/
3 exp Anoxia/
4 exp Hypoxia, Brain/
5 ((brain or head or intracran* or cerebr* or cerebellar or brainstem or vertebrobasilar) adj3 (injur* or infarc* or isch?em* or thrombo* or apoplexy or emboli* or h?emorrhag* or h?ematoma* or aneurysm* or anoxi* or hypoxi*)).ab,ti.
6 (encephaliti* or mening*).ab,ti.
7 1 or 2 or 3 or 4 or 5 or 6
8 review$.tw.
9 randomized controlled trial.pt.
10 random$.tw.
11 control$.tw.
12 intervention$.tw.
13 evaluat$.tw.
14 or/8-13
15 Fee-for-Service Plans/
16 Fees, Medical/
17 Insurance, Health, Reimbursement/
18 (fee-for-service or fee for service).tw.
19 ((client or patient or insur$) adj (charge or pay$ or bill or cost or contribut$ or fee)).tw.
20 Continuity of Patient Care/
21 Patient Care Team/
22 Delivery of Health Care, Integrated/
23 ((seamless or integr$ or contin$) adj (care or rehab$ or therap$ or intervention)).tw.
24 Case Management/
25 Managed Care Programs/
26 Patient-Centered Care/
27 (continuity or continuum or collaborative or shared care or case manage$ or care plan).tw.
28 Care coordination.mp.
29 exp Consumer Participation/
30 ((consumer$ or patient$ or stakeholder$ or user$ or lay or citizen$ or public or client$) adj (particip$ or involv$ or represent$ or collaborat$ or consult$ or contribut$ or engag$ or deliberat$ or dialogue or opinion?)).tw.
31 Quality of Health Care/
32 Quality Assurance, Health Care/
33 Critical Pathways/
34 Clinical Protocols/
35 Clinical Governance/
36 (quality adj (assurance or monitor$ or improv$)).tw.
37 ICF.mp. or (International Classification for Functioning, Disability and Health).ab. [mp=ti, ab, sh, hw, tn, ot, dm, mf, dv, kw, nm, kf, ps, rs, an, ui, tc, id, tm]
38 or/15-37
39 7 and 14 and 38
40 limit 39 to journal article
41 limit 40 to english language
42 limit 41 to human
43 limit 42 to yr="1980 -Current"
